# Rethinking the Role of Radiation Therapy in the Treatment of Unresectable Hepatocellular Carcinoma: A Data Driven Treatment Algorithm for Optimizing Outcomes

**DOI:** 10.3389/fonc.2019.00345

**Published:** 2019-06-18

**Authors:** Mutlay Sayan, Nikhil Yegya-Raman, Stephanie H. Greco, Bin Gui, Andrew Zhang, Anupama Chundury, Miral S. Grandhi, Howard S. Hochster, Timothy J. Kennedy, Russell C. Langan, Usha Malhotra, Vinod K. Rustgi, Mihir M. Shah, Kristen R. Spencer, Darren R. Carpizo, John L. Nosher, Salma K. Jabbour

**Affiliations:** ^1^Department of Radiation Oncology, Rutgers Cancer Institute of New Jersey, Rutgers Robert Wood Johnson Medical School, Rutgers University, New Brunswick, NJ, United States; ^2^Division of Surgical Oncology, Rutgers Cancer Institute of New Jersey, Rutgers Robert Wood Johnson Medical School, Rutgers University, New Brunswick, NJ, United States; ^3^Division of Medical Oncology, Rutgers Cancer Institute of New Jersey, Rutgers University, New Brunswick, NJ, United States; ^4^Division of Gastroenterology and Hepatology, Rutgers Robert Wood Johnson Medical School, Rutgers University, New Brunswick, NJ, United States; ^5^Division of Surgical Oncology, Department of Surgery, Emory University School of Medicine, Atlanta, GA, United States; ^6^Department of Radiology, Rutgers Robert Wood Johnson Medical School, Rutgers University, New Brunswick, NJ, United States

**Keywords:** hepatocellular carcinoma, radiation therapy, transcatheter arterial chemoembolization, transarterial embolization, systemic therapy

## Abstract

Hepatocellular carcinoma (HCC) is the second most common cause of cancer death worldwide, with a majority of HCC patients not suitable for curative therapies. Approximately 70% of initially diagnosed patients cannot undergo surgical resection or transplantation due to locally advanced disease, poor liver function/underlying cirrhosis, or additional comorbidities. Local therapeutic options for patients with unresectable HCC, who are not suitable for thermal ablation, include transarterial embolization (bland, chemoembolization, radioembolization) and/or external beam radiation therapy (EBRT). Regarding EBRT specifically, technological advancements provide a means for safe and effective radiotherapy delivery in a wide spectrum of HCC patients. In multiple prospective studies, EBRT delivery in a variety of different fractionation schemes or in combination with transcatheter arterial chemoembolization (TACE) demonstrate improved outcomes, particularly with combination therapy. The Barcelona Clinic Liver Cancer classification provides a framework for treatment selection; however, given the growing complexity of treatment strategies, this classification system tends to simplify decision-making. In this review, we discuss the current literature regarding unresectable HCC and propose a modified treatment algorithm that emphasizes the role of radiation therapy for Child-Pugh score A or B patients with ≤3 nodules measuring >3 cm, multinodular disease or portal venous thrombosis.

## Introduction

Hepatocellular carcinoma (HCC) is the second most common cause of cancer death worldwide (1). While the incidence of HCC is highest in Asia, the rate has been increasing significantly in North America (2). HCC can result in patients with liver cirrhosis, and known major risk factors for cirrhosis include viruses [chronic hepatitis B virus (HBV) and hepatitis C virus (HCV)], toxins (e.g., alcohol, tobacco, and aflatoxins), and metabolic disorders (e.g., nonalcoholic steatohepatitis, and diabetes) and other conditions, such as hereditary hemochromatosis (3). The American Association for the Study of Liver Diseases (AASLD) recommends surveillance of patients with cirrhosis using ultrasound, with or without alpha-fetoprotein (AFP), every 6 months (4). Patients with HCC are often asymptomatic at the time of diagnosis leading to a delay in diagnosis for patients not being screened for HCC in the setting of viral hepatitis infection. Classic imaging characteristics of arterial enhancement and venous or delayed-phase washout of lesions >1cm in patients with cirrhosis or chronic HBV are considered by some as diagnostic for HCC even in the absence of histologic confirmation (5).

Local control (LC) is the most important prognostic factor for HCC, because up to 92% of deaths can be directly correlated to local progression leading to liver failure rather than distant metastases (6, 7). While liver transplantation or surgical resection remains the principal curative option for patients with HCC, only 30% are suitable for this therapy. Patients are often deemed non-surgical candidates due to locally advanced disease, poor liver function, additional comorbidities, and/or poor performance status (8). For HCC patients who are non-surgical candidates, an alternative curative therapeutic option is radiofrequency ablation (RFA), which has optimal outcomes in tumors <3 cm that are primarily located away from major blood vessels, bile ducts, and abdominal organs (9). Some unresectable HCC patients are also not candidates for RFA due to location of the tumor, intrahepatic bile duct dilation, and volume of disease; for these patients other local therapies include transcatheter arterial bland embolization, chemoembolization (TACE), radioembolization (TARE) with Yttrium-90 (^90^Y) microspheres, and external beam radiation therapy (EBRT). Inoperable lesions for which local ablation is not possible are treated with TACE since non-randomized studies suggest it may improve survival compared to best supportive care (10, 11). Historically, liver EBRT was not employed due to risk of radiation-induced liver disease (12, 13). However, modern imaging techniques, advances in EBRT planning and delivery, and improvements in biological understanding of radiation dose tolerances to liver parenchyma have led to reconsideration of EBRT in the context of other local treatment options. Moreover, with a better understanding of the dose-volume effects of partial liver radiation and utilization of advanced radiation technology, severe toxicity rates following EBRT are now less than 10% (14).

The Barcelona Clinic Liver Cancer (BCLC) staging classification combines tumor number, size, extent of spread, performance status, and Child Pugh (CP) score to provide treatment recommendations for patients with HCC (Table 1) (15, 16). While the BCLC is the most commonly used treatment algorithm for newly diagnosed HCC, it may inadequately reflect therapeutic advances and simplify decision making, particularly in patients with BCLC Stage B or C disease. For example, the algorithm does not provide treatment recommendations for patients with ≤3 nodules measuring >3 cm, a common presentation in the era of advanced imaging techniques. Furthermore, in light of growing prospective data evaluating the role of modern radiation therapy (RT) for HCC, EBRT has emerged as a potential treatment option in select patients with BCLC Stage B and C disease and EBRT is not incorporated into this algorithm.

**Table 1:** Barcelona clinic liver cancer staging system^*^.

**Stage**	**Tumor extent**	**PS**	**Child-Pugh score**
0	Single nodule ≤2 cm	0	A
A	Single or up to 3 nodules <3 cm	0	A or B
B	Multinodular	0	A or B
C	Spread to blood vessels, lymph nodes or other organs	1 or 2	A or B
D	–	3 or 4	C

*PS, performance status*.

* *Patient's overall stage is determined by whichever of the three categories (tumor extent, PS or Child-Pugh score) is most advanced*.

**Figure 1:**
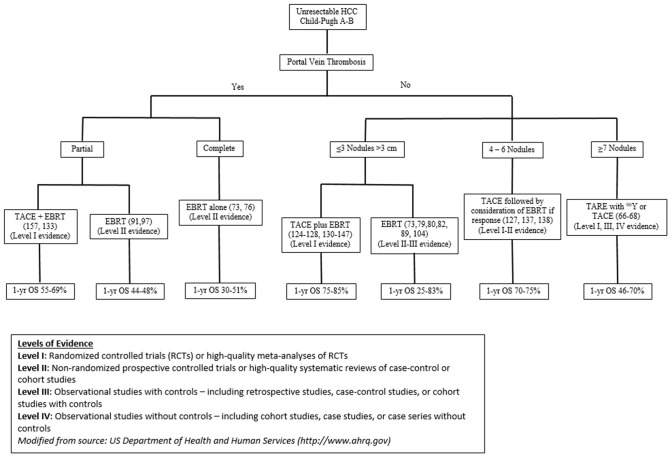
Data driven Rutgers treatment algorithm for optimizing outcomes for unresectable HCC patients.

Given this constantly evolving treatment paradigm, herein, we evaluate the published data on local therapeutic options for unresectable HCC and propose a functional treatment algorithm for CP-A or B patients with ≤3 nodules measuring >3 cm, multinodular disease, or portal venous thrombosis (PVT) (Figure 1).

## TACE

TACE is a commonly used treatment modality in patients with HCC who are deemed poor candidates for curative treatment with surgery or RFA. According to a recent Surveillance, Epidemiology, and End Results (SEER) database analysis, TACE is the most common initial therapy utilized in the US (64%) among patients who receive treatment for HCC (17). This treatment involves the intra-arterial injection of chemotherapeutic agents followed by obstruction of selective hepatic arterial inflow with embolizing particles. The purpose of this procedure is to increase tumor cell exposure to cytotoxic agents and selectively tamponade the blood supply to the tumor or affected liver lobe. The resultant stasis of blood flow and hypoxia induces vascular endothelial growth factor (VEGF) secretion, which increases vessel permeability and results in higher intra-hepatic chemotherapy deposition (18). TACE with drug-eluting beads (DEB-TACE) utilizes embolizing particles to both embolize the hepatic artery and carry the cytotoxic agents. The most commonly employed chemotherapeutic agents are mitomycin-C and doxorubicin.

Patient selection for TACE is an important step to avoid treatment-related adverse events. Ideal candidates present with Eastern Cooperative Oncology Group (ECOG) performance status ≤2, preserved liver function (CP-A), and tumors measuring <10 cm without portal vein thrombosis (PVT). Select patients with impaired liver function (CP-B) and mildly impaired performance status can be treated with TACE; however, the incidence of treatment-related toxicities including abdominal pain, nausea, and vomiting increases in this patient population (19). Contraindications to TACE include an ECOG performance status >2, advanced cirrhosis (CP-C), >50% replacement of the liver by tumor, PVT, renal insufficiency (creatinine ≥2 mg/dl or creatinine clearance <30 ml/min), bilirubin levels >3 mg/dL, macroscopic vascular invasion, extrahepatic disease, bile duct occlusion, and comorbidities involving compromised organ function, such as active cardiovascular disease (20–25).

Multiple studies have demonstrated, when compared to best supportive care, TACE improves survival in HCC patients with a wide range of disease states including CP-A and B, tumor size measuring <14 cm, and multinodular disease (Table 2) (10, 11, 26–30). In order to further improve the response to TACE, combination treatments with systemic and/or other locoregional therapies have been investigated in recent years (31–40).

**Table 2:** Studies of transcatheter arterial chemoembolization vs. best supportive treatment for hepatocellular carcinoma.

**References**	**Study design**	***N***	CPS A/B/C (^*^Okuda I/II/III) (%)	**Tumor size**	**Follow up**	**Overall survival**	***p***
						**TACE vs. BST**	
Pelletier et al. (26)	Prospective	42	29/52/19^*^	34-41% of the liver	1 yr	6 mo 33% vs.53%	NS
						12 mo 24% vs.31%	
Groupe d'Etude (27)	Prospective	96	90/10/0^*^	10% with ≥ 50% of the liver	4 yrs	1 yr 62% vs.44%	NS
						2 yr 38% vs.36%	
Mabed et al. (28)	Prospective	100	69/31/0	NR	1 yr	38 wks vs.32 wks	0.08
Pelletier et al. (29)	Prospective	73	77/23/0	NR	2 yrs	1 yr 51% vs.55%	0.77
Lo et al. (11)	Prospective	79	47/53/0^*^	7 cm (4–14)	3.5 yrs	1 yr 57% vs.32%	0.002
						2 yr 31% vs.11%	
						3 yr 26% vs.3%	
Llovet et al. (10)	Prospective	112	69/31/0	4.9 cm (4–5.8)	21 mo	1 yr 82% vs.63%	0.009
						2 yr 63% vs.27%	
						3 yr 29% vs.17%	
Doffoel et al. (30)	Prospective	123	71/29/0	12% with ≥ 50% of the liver	12 mo	1 yr 51% vs.46%2 yr 25% vs.22%	0.68

*CPS, Child-Pugh score; TACE, transarterial chemoembolization; BST, best supportive treatment; NR, not reported; NS, not significant*.

* *Okuda stage*.

### TACE Plus Molecularly Targeted Therapy

Treatment with TACE can lead to the promotion of tumorigenesis and angiogenesis (41), which may partially explain the limited long-term benefit of this therapy. Thus, in an attempt to improve the efficacy of TACE, studies have combined this treatment with concurrent systemic targeted therapies (Table 3) (31–40). One such targeted therapy is sorafenib, a small molecule inhibitor of several tyrosine protein kinases (TKI) including vascular endothelial growth factor receptors (VEGFRs)-1, 2, and 3 and platelet-derived growth factor receptor β (PDGFR-β) (43, 44). In preclinical studies, sorafenib demonstrated antiproliferative activity in malignant hepatic cell lines by decreasing tumor angiogenesis and tumor-cell signaling as well as increasing tumor-cell apoptosis (45). Given these findings, in a multicenter randomized phase III trial, sorafenib demonstrated improved median overall survival (OS) when compared to placebo (10.7 months vs. 7.9 months; *P* < 0.001) in patients with CP-A advanced HCC (46). Furthermore, in cell culture models, sorafenib reduced the susceptibility of hepatocytes to HCV infection via anti-VEGF activity (47, 48) and directly inhibited HCV replication via non-structural HCV replicon protein NS5A interaction with C-Raf (49). In light of this preclinical information, sorafenib is also thought to be more effective in hepatitis-related HCC (50).

**Table 3:** Studies of transcatheter arterial chemoembolization plus systemic therapy vs. transcatheter arterial chemoembolization alone for hepatocellular carcinoma.

**References**	**Study design**	***N***	**CPS A/B/C (%)**	**Tumor size**	**Follow-up**	**Systemic therapy**	**Overall Survival**	***p***
							**TACE + ST vs. TACE alone**	
Sansonno et al. (31)	Prospective	80	100/0/0	7.4 vs.6.9 cm	21 mo	sorafenib	TTP 9 mo vs.5 mo	0.001
Kudo et al. (32)	Prospective	458	100/0/0	≤7 cm (max)	3 yrs	sorafenib	1 yr 95% vs.94%	NS
							2 yr 72% vs.74%	
Yao et al. (33)	Prospective	150	84/16/0	NR	14 mo	sorafenib	22 mo vs.12 mo	<0.001
Bai et al. (34)	Prospective	304	77/23/0	NR	21 wks	sorafenib	1 yr 32% vs.24%	0.009
Britten et al. (35)	Prospective	30	93/7/0	6.5 vs.7.4 cm	5 yrs	bevacizumab	61 mo vs.49 mo	0.21
Pinter et al. (37)	Prospective	32	69/31/0	≤15 cm (max)	46 mo	bevacizumab	1 yr 31% vs.55%	0.195
Wang et al. (36)	Prospective	125	85/15/0	NR	40 mo	arsenic trioxide	1 yr 93% vs.64%	<0.05
							2 yr 76% vs.51%	
Kudo et al. (38)	Prospective	502	95/5/0	<10 cm in 77%	3 yrs	brivanib	1 yr 74% vs.68%	0.528
							2 yr 52% vs.54%	
Inaba et al. (39)	Prospective	101	84/16/0	≤8 cm (max)	3 yrs	TSU-68	PFS 157 vs.122 days	0.054
Lencioni et al. (42)	Prospective	307	100/0/0	NR	6 mo	sorafenib	TTP 5.6 mo vs.5.5 mo	0.072

*CPS, Child-Pugh score; TACE, transarterial chemoembolization; ST, systemic therapy; NR, not reported; NS, not significant; TTP, time to progression; PFS, progression-free survival*.

Given the mechanisms of action of both sorafenib and TACE, there is growing support for this therapeutic combination to take advantage of a possible synergistic effect. TACE increases the concentration of angiogenic growth factors such as VEGF and insulin-like growth factor-2 (IGF-2), which may contribute to disease progression (51), while sorafenib inhibits angiogenic growth factors to prevent progression. Based on this rationale, the combination of TACE with sorafenib has been investigated in a number of studies (32, 42, 52). Lencioni et al. randomized 307 intermediate stage HCC patients to sorafenib plus drug eluting bead (DEB)-TACE vs. placebo plus DEB-TACE (42). All patients had no evidence of macrovascular invasion or extrahepatic spread, were CP-A, and had an ECOG performance status of 0. There was no difference in median time-to-tumor progression (TTP) between the two groups (5.6 months vs. 5.5 months, hazard ratio (HR) 0.797, *p* = 0.072). Additionally, the overall response rates for patients receiving sorafenib vs. placebo were 55.9% and 41.3%, respectively, and the disease control rates were 89.2% and 76.1%, respectively. Kudo et al. randomized 458 HCC patients with CP-A and tumors ≤3 cm to sorafenib plus TACE or TACE alone. Similarly, there were no differences in OS at 1 year (95% vs. 94%) or 2 years (72% vs. 74%) (32). While Lencioni et al. and Kudo et al. reported combination therapy did not impact OS, smaller prospective studies evaluating the same combination therapy reported opposite results with improved survival (31, 33).

Therefore, TACE has been combined with multiple targeted agents including sorafenib, but to date, this combination therapy has not led to a meaningful increase in survival (Table 3) (35–38, 40). While sorafenib is considered first-line systemic therapy after failure of liver-directed therapies, a new multikinase inhibitor, lenvatinib, has emerged as a new alternative first-line treatment option (53). In the randomized phase III REFLECT trial, lenvatinib demonstrated a comparable OS (median, 13.6 vs. 12.3 months, HR 0.92, 95% CI 0.79–1.06) and improved TTP (median, 8.9 vs. 3.7 months, HR 0.63, 95% CI 0.53–0.73) over sorafenib. In the phase I/II CheckMate 040 trial, a PD-1 inhibitor, nivolumab, demonstrated a 20% objective response (HR 95% CI 15–26) in patients with advanced HCC and is now approved as second-line therapy following prior sorafenib (54). More recently, in the phase III CLESTIAL trial, cabozantinib has been shown to improve median OS compared with placebo after progression on sorafenib (10.2 vs. 8 months, *P* = 0.005) (55). Given the encouraging results of these studies, combination of these new agents with TACE should be investigated in prospective studies.

Radiosensitization with systemic therapy is the principle underlying many treatment regimens for solid malignancies and has gained interest for HCC in recent years. RT has multiple effects on the tumor microenvironment and the immune system, including cytokine and antigen release leading to increased immune cell infiltrate (56). The potential increase in toxicity with using combination therapy remains the main concern as several clinical studies (NCT03203304 and NCT03482102) are currently evaluating the optimal combination strategies with RT.

### TACE Plus RFA

Radiofrequency ablation (RFA) plus TACE can also be utilized in HCC patients. RFA is considered an effective treatment for tumors <3 cm by conducting high-energy electrical current or microwaves into the target lesion which then leads to tumor tissue necrosis (57). Reported LC rates are as high as 90%; however, this decreases significantly with increasing tumor size as well as close proximity to major vessels due to a heat-sink effect (58, 59). The heat-sink effect is a phenomenon that occurs when flowing hepatic blood causes a cooling effect, thereby reducing the ablation volume. Based on the theory that performing TACE before RFA may allow retention of thermal energy within the tumor environment by decreasing blood flow, studies compared RFA alone to TACE plus RFA and demonstrated improved survival for the latter in patients with HCC measuring <3 cm (60). However, the survival benefit decreases in tumors >3 cm. Lin et al. randomized 62 patients with HCC to either TACE plus RFA or RFA alone from 2006 to 2010 (61). Patients were CP-A or B and had ≤ 3 tumors measuring 3–5 cm with no evidence of extrahepatic tumor metastasis or macrovascular invasion. The 1-, 2-, and 3-year local tumor progression rates in the TACE plus RFA group (12.5%, 18.75%, and 18.75%) were significantly lower than in the RFA alone group (16.7%, 30%, and 36.6%, *P* = 0.047). However, 1-, 2-, and 3- year OS rates remained similar between the two treatment groups (90.6% vs. 83.3%, 72% vs. 56.75%, and 53.1% vs. 23.3%, *P* = 0.176). Given the improved prognosis with combination therapy in patients with small HCCs, TACE plus RFA may be considered in CP-A or B patients with ≤3 tumors measuring <3 cm.

### Transarterial Radioembolization (TARE) With Yttrium-90 (^90^Y)

TARE with ^90^Y involves the injection of β-emitting ^90^Y loaded glass matrices or resin microspheres into the hepatic artery which leads to delivery of concentrated radiation to the tumor. The radioisotope ^90^Y is a pure β-radiation emitter with a half-life of 64.2 h, an average energy of 0.94 MeV, and an average penetration range in tissue of 2.5 mm (62–64). Absolute contraindications for ^90^Y radioembolization include significant intractable clinical ascites, bleeding diathesis, severe portal hypertension with hepatofugal flow, or severe peripheral vascular disease that would preclude arterial catheterization (65). Moreno-Luna et al. compared unresectable HCC patients treated with TARE (*n* = 61, 87% CP-A, 69% multinodular, and mean tumor size 6 cm, range 2–9 cm) in a non-randomized study to those treated with TACE (*n* = 55, 80% CP-A, 42% multinodular, and mean tumor size 6 cm, range 2–10 cm) between 2005 and 2008 (66). While the complete tumor response rate was higher with TARE (12% vs. 4%, *p* = 0.17), there was no difference in median OS between the two groups (15.0 months for TARE vs. 14.4 months for TACE; *p* = 0.47). Furthermore, TARE was more likely to induce fatigue (*p* = 0.003) but less likely to cause fever (*p* = 0.02). Salem et al. also prospectively compared unresectable HCC patients treated with TARE (*n* = 123, 54% CP-A, 55% multinodular, and mean tumor size 5 cm, range 2–7 cm) to those treated with TACE (*n* = 122, 55% CP-A, 53% multinodular, and mean tumor size 3 cm, range 2–6 cm) (67). They found median TTP was longer following TARE (13.3 months vs. 8.4 months, *p* = 0.046) but median OS did not differ significantly between the two groups (17.4 months vs. 20.5 months, respectively, *p* = 0.232). Additionally, abdominal pain and increased transaminase activity were more common with TACE (*p* < 0.05). In the phase III SIRveNIB trial, Chow et al. randomized 360 HCC patients (90% CP-A and 24% with tumor size >50% of liver) to TARE or sorafenib (68). While there was no difference in OS (HR, 1.1; 95% CI, 0.9 to 1.4; *p* = 0.36), there was an improvement in response rate with TARE (16.5% vs. 1.7%, *P* < 0.001), as well as reduced grade ≥3 toxicity compared with sorafenib (27.7% vs. 50.6%, *P* < 0.001). In light of this data, ^90^Y radioembolization is considered a viable treatment option for patients with multinodular HCC, but sorafenib remains a standard of care.

## Radiation Therapy

Technological advances in EBRT such as CT-based treatment planning, management of respiratory motion, understanding of treatment dose distributions, delineation of organs at risk (OAR), and the transition from whole liver irradiation (WLI) to more conformal/dose-escalated treatment regimens have provided the opportunity to offer EBRT safely and effectively. As such, the use of EBRT in patients with HCC has increased in recent years (69). RT techniques, including 3D-conformal radiotherapy (3D-CRT), intensity modulated radiotherapy (IMRT), stereotactic-body radiotherapy (SBRT), and proton beam radiotherapy (PBT), have allowed for the delivery of higher RT doses to tumor volumes compared to historical WLI, and in turn, may have resulted in improved outcomes when compared to other local therapies for HCC (58, 70, 71).

Different RT techniques have been used for a wide range of patients and within various HCC subgroups. Nevertheless, we must note that the use of RT for patients with CP-C disease is very limited. Furthermore, the data on the use of RT for patients with CP-B disease is still unsettled, as a smaller proportion of patients with CP-B disease were enrolled in clinical trials (Tables 4–**7**). Therefore, use of RT in this subgroup of patients should be carried out after multi-disciplinary evaluation with individualized treatment for each patient.

**Table 4:** Studies of 3D-CRT and IMRT for hepatocellular carcinoma.

References	Study design	Modality	*N*	Tumor size	CPS A/B/C (%)	Radiation therapy dose	Follow-up	Response (C/P)	Overall survival
Cheng et al. (72)	Prospective	3D-CRT	13	15 cm (6–25)	69/31/0	40–60 Gy @ 1.8–2 Gy/fx	40 mo	58%	1 yr 100%
Liu et al. (73)	Prospective	3D-CRT	44	NR	73/27/0	40–60 Gy @ 1.8 Gy/fx	8 mo	61%	1 yr 61% 2 yr 40%
Mornex et al. (74)	Prospective	3D-CRT	27	3.2 cm (1–5)	59/41/0	66 Gy @2 Gy/fx	29 mo	92%	NR
Kim et al. (75)	Retrospective	3D-CRT	70	7.5 cm (2–17)	80/20/0	44–54 Gy @ 2–3 Gy/fx	9 mo	54%	1 yr 43% 2 yr 18%
Kim et al. (76)	Prospective	IMRT	35	NR	80/20/0	45–60 Gy @ 4.6–6 Gy/fx	13 mo	52%	1 yr 51%
									2 yr 22%
Chi et al. (77)	Prospective	IMRT	23	NR	65/35/0	52.5 Gy @ 2.5–4.5 Gy/fx	16 mo	74%	1 yr 70%
McIntoch et al. (78)	Retrospective	IMRT	20	9 cm	55/45/0	30–50 Gy @ 2.5 Gy/fx	NR	66%	1 yr 75%
				(1.3–17)					2yr 50%
Kang et al. (79)	Retrospective	IMRT	27	11 cm (8–18)	70/30/0	45–64.8 Gy @ 1.8 Gy/fx	5 mo	44%	5 mo (median)
Kong et al. (80)	Retrospective	IMRT	22	4.4 cm	68/32/0	30–60 Gy @ 1.8–4.5 Gy/fx	14 mo	73%	1 yr 86%
				(0.9–16)					2 yr 69%
Huang et al. (81)	Retrospective	IMRT	38	4.6 cm	71/29/0	46–72 Gy @ 1.8–2.4 Gy/fx	17 mo	53%	1 yr 56%
				(2.5–17)					2 yr 32%

*CPS, Child-Pugh score; 3D-CRT, 3-dimensional conformal radiotherapy; IMRT, intensity-modulated radiotherapy; NR, not reported; C/P, complete/partial*.

### Conformal Radiotherapy (CRT)

3D-CRT and IMRT improve the effectiveness of EBRT by increasing the RT dose to the tumor while simultaneously reducing the RT dose to the surrounding normal liver parenchyma when compared to WLI (Table 4) (72–81). Historically, EBRT techniques used for HCC consisted of 2-dimentional planning. Given the associated toxicities of this WLI technique, it was not considered clinically beneficial owing to the sub-therapeutic dose of RT delivered to the tumor.

With the advancement of RT delivery to 3D techniques, the normal liver parenchyma could be from spared the high dose exposure while the dose to the tumor itself could be increased. The ability to escalate dose has been a significant improvement since studies have demonstrated that higher RT dose to the tumor correlates with better OS. Seong et al. treated 158 patients with HCC (74% CP-A, 26% CP-B, 51% PVT, 75% tumor size <10 cm, and 25% tumor size >10 cm) with a dose of 25.2–60 Gy in 1.8 Gy per fraction (142). The median OS in patients treated with <40 Gy, 40–50 Gy, and >50 Gy were 6 months, 8 months, and 13 months, respectively. On multivariate analysis, greater RT dose to the tumor was the only significant factor associated with survival (p = 0.01). Other studies also demonstrated that a total dose of >40–50 Gy in standard fractionation led to a higher response or survival rate (73, 143, 144). Larger radiotherapy doses can often more readily be delivered to smaller tumors in locations where nearby organs are not abutting the tumor. However, the tolerance dose of the normal liver parenchyma, especially in the setting of poor baseline liver function, often limits the use of higher doses of EBRT in the setting of HCC.

IMRT is another conformal RT technique that allows delivery of a higher RT dose when compared to 3D-CRT which may further improve OS without increasing the risk of radiation-induced liver disease (RILD) in patients with ≤3 tumor nodules measuring >3 cm and/or PVT. IMRT uses inverse treatment planning which modulates the intensity of multiple beams to gain a desired target coverage while minimizing the dose to normal structures. Early dosimetric studies comparing IMRT to 3D-CRT suggested that IMRT improves planning target volume (PTV) coverage while maintaining normal tissue tolerances (145). Yoon et al. retrospectively reviewed 187 patients with HCC and CP-A treated with 3D-CRT (*n* = 122; median fractional and total dose: 1.8 Gy and 45 Gy, respectively) or IMRT (*n* = 65; median fractional and total dose: 2.5 Gy and 50 Gy, respectively) from 2006 to 2011. Median tumor size (9 cm vs. 10 cm, *p* = 0.779) and ECOG PS ≤1 (44% vs. 40%, *p* = 0.557) were similar in both groups and 74% had ≤3 tumor nodules. Patients treated with IMRT had significantly higher 3-year OS (33.4% vs. 13.5 %, *P* < 0.001), progression-free survival (PFS) (11.1% vs. 6.0%, *P* = 0.004), and in field-failure-free survival rates (46.8% vs. 28.2%, *P* = 0.007) when compared to patients treated with 3D-CRT; no difference in RILD was demonstrated (*P* = 0.716) (146). Similar results were reported by Hou et al. in 118 HCC patients with portal vein and/or inferior vena cava tumor thrombi (81% CP-A and 19% CP-B) (147). Higher RT doses were delivered when IMRT was utilized compared to 3D-CRT (average dose 57.86 ± 7.03 Gy vs. 50.88 ± 6.60 Gy, *P* ≤ 0.001). Additionally, median OS was significantly higher in patients treated with IMRT compared to 3D-CRT (15.47 months vs. 10.46 months, *P* = 0.005) while the overall toxicity was similar between the two groups (grade 3 toxicity 5% vs. 2%, *P* = 0.786). While robust prospective data are lacking, dose escalation with IMRT is considered as a treatment modality in HCC patients with CP-A/B and ≤3 tumor nodules measuring >3 cm and/or PVT.

### SBRT

SBRT is a type of EBRT that delivers an highly conformal high dose of RT to a target in 1–5 fractions. One of the first series evaluating SBRT for HCC was described by Blomgren et al. in 1995 (148); since then, this technique has demonstrated excellent outcomes in numerous trials/studies despite often being utilized in patients unsuitable for other therapies believed to have a poor prognosis (Table 5) (82–103).

**Table 5:** Studies of stereotactic body radiation therapy for hepatocellular carcinoma.

References	Study design	*N*	Tumor size	CPS A/B/C (%)	Radiation therapy dose	Follow-up	Local control	Overall survival
Scorsetti et al. (82)	Prospective	43	4.8 cm	53/47/0	48–75 Gy in 3 fx or 36–60 Gy in 6 fx	8 mo	6 mo 94%	6 mo 91%
			(1–12.5)				1 yr 85%	1 yr 78%
							2 yr 64%	2 yr 45%
Méndez-Romero et al. (83)	Prospective	8	3.2 cm (0.5–7.2)	63/37/0	25–38 Gy in 3–5 fx	13 mo	1 yr 75%	1 yr 75%
Bujold et al. (84)	Prospective	102	7.2 cm (1.4–23)	100/0/0	24–54 Gy in 6 fx	31 mo	1 yr 87%	1 yr 55%
Kang et al. (85)	Prospective	47	2.9 cm (1.3–7.8)	87/13/0	42–60 Gy in 3 fx	165 mo	2 yr 95%	2 yr 69%
Lasley et al. (86)	Prospective	59	33.6 cc (2–107)	64/36/0	40–48 Gy in 3-5 fx	33 mo	3 yr 91%	3 yr 61%
Takeda et al. (87)	Prospective	90	≤4 cm	91/8/0	35–40 Gy in 5 fx	42 mo	3 yr 96%	3 yr 67%
Kwon et al. (88)	Prospective	42	15 cc	90/10/0	30–39 Gy in 3 fx	49 mo	1 yr 72%	1 yr 93%
			(3–81)				3 yr 68%	3 yr 87%
Louis et al. (89)	Prospective	25	4.5 cm	88/12/0	45 Gy in 3 fx	24 mo	1 yr 95%	1 yr 79%
			(1.8–10)				2 yr 95%	2 yr 52%
Tse et al. (90)	Prospective	31	173 mL (9–1913)	NR	24–55 Gy in 6 fx	18 mo	1 yr 65%	1 yr 51%
Takeda et al. (91)	Prospective	16	14 cc (3.4–72)	88/12/0	35–50 Gy in 5–7 fx	20 mo	1 yr <90%	NR
Seo et al. (92)	Prospective	38	All <10 cm	89/11/0	33–57 Gy in 3–4 fx	27 mo	1 yr 79%	1 yr 68%
							2 yr 66%	2 yr 61%
Kim et al. (93)	Prospective	18	1.9 cm	100/0/0	36–60 Gy in 4 fx	28 mo	1 yr 78%	1 yr 94%
			(1–3.3)				2 yr 71%	2 yr 69%
Price etl al. (94)	Prospective	26	All ≤ 6 cm	54/46/0	24–48 Gy in 3–5 fx	13 mo	73% C/P response	1 yr 77%
								2 yr 60%
Su et al. (95)	Retrospective	132	3 cm	86/14/0	42–46 Gy in 3–5 fx or 28–30 Gy in 1 fx	21 mo		1 yr 94%
			(1.1–5)				1 yr 91%	2 yr 82%
							2 yr 84%	3 yr 58%
								5 yr 36%
Yamashita et al. (96)	Retrospective	79	2.7 cm (0.6–7)	85/11/1	40–60 Gy in 4–10 fx	21 mo	21 mo 80%	2 yr 53%
Bibault et al. (97)	Retrospective	75	3.7 cm	89/11/0	45 Gy in 3 fx	10 mo	1 yr 90%	1 yr 79%
			(3–4.4)				2 yr 90%	2 yr 50%
Andolino et al. (98)	Retrospective	60	3.2 cm (1–6.5)	60/40/0	44 Gy in 3 fx	27 mo	2 yr 90%	2 yr 67%
Huang et al. (99)	Retrospective	36	4.4 cm (1–12)	78/19/3	25–48 Gy in 4–5 fx	14 mo	2 yr 75%	2 yr 73%
Bae et al. (100)	Retrospective	35	131 mL	91/9/0	30–60 Gy in 3–5 fx	14 mo	1 yr 69%	1 yr 52%
			(21—2189)				3 yr 51%	3 yr 21%
Sanuki et al. (101)	Retrospective	161	2.4 cm (0.8–4.9)	74/26/0	35–40 Gy in 5 fx	28 mo	2 yr 92%	2 yr 80%
Huertas et al. (102)	Retrospective	77	2.4 cm	82/18/0	15–60 Gy in 3 fx	12 mo	1 yr 99%	1 yr 82%
			(0.7–6.3)				2 yr 99%	2 yr 57%
Kimura et al. (103)	Retrospective	65	1.6 cm (0.5–5.4)	78/22/0	48 Gy in 4 fx	26 mo	2 yr 100%	2 yr 76%

*CPS, Child-Pugh score; NR, not reported; C/P, complete/partial*.

Although there are no phase III data yet, growing retrospective as well as prospective evidence support promising outcomes with SBRT and its use as an alternative HCC therapy. Yuan et al. retrospectively compared 48 patients with HCC treated with SBRT (*n* = 22, 88% CP-A, 12% CP-B, median tumor size 4.3 cm, median dose 45 Gy, range 39–54 Gy in 3–8 fractions) or microscopic complete resection (*n* = 26, CP-A, 26% CP-B median tumor size 4.6 cm) from 2006 to 2011 and found no significant difference in OS or PFS between the two cohorts (149). Sapir et al. reported the outcomes of 209 patients with HCC treated with SBRT (*n* = 125, ≤2 tumor nodules, median tumor size 2.4 cm, range 0–20.8 cm, median CP-score 6, range 5–9) vs. TACE (*n* = 84, ≤2 tumor nodules, median tumor size 2.7 cm, range 0.7–15 cm, median CP-score 6, range 5–9) (70). The 1- year and 2- year LC rates favored SBRT (97% and 91%, respectively) when compared to TACE (47% and 23%, respectively; HR 66.5, *p* < 0.001). Wahl et al. retrospectively reported the outcomes from 224 patients with HCC (median tumor size 2.2 cm; range 0–10 cm) treated with SBRT (*n* = 63, 69% CP-A, 29% CP-B, 92% ≤2 tumor nodules, and median dose 27 to 60 Gy in 3–5 fractions) or RFA (*n* = 161, 50% CP-A, 18% CP-B, and 89% ≤2 tumor nodules) from 2004 to 2012 (58). One-year liver specific PFS after SBRT compared to RFA was 97.4% vs. 83.6%, and 2-year OS rates were 46% vs. 53%, respectively. For tumors ≥2 cm, LC with RFA was significantly lower compared to SBRT (HR, 3.35; 95% CI, 1.17 to 9.62; *p* = 0.025). There was no difference in acute grade ≥3 toxicities between the RFA and SBRT groups (11% vs. 5%, *p* = 0.31). Mendez-Romero et al. reported the first prospective outcomes of SBRT for HCC in 2006. Eight HCC patients (median tumor size 3.2 cm and 63% with CP-A) who were ineligible for other local therapies received SBRT (83); tumors <4 cm received 37.5 Gy in 3 fractions and those ≥4 cm received 25 Gy in 5 fractions. One-year LC and OS rates were both 75%. Local failure was seen in 4 patients that were in the 25 Gy treatment arm. Kang et al. reported the efficacy of SBRT as a local salvage treatment after incomplete response to TACE (85). Forty-seven patients with HCC (median tumor size 2.9 cm, 87% CP-A, and 98% ≤2 tumor nodules) were treated with a RT dose up to 60 Gy in 3 fractions (42–60 Gy) 1 to 2 months post TACE. The 2-year LC, OS, and PFS rates were 95%, 69%, and 34%, respectively. SBRT was well tolerated, with CP-class worsening from A to B in 13% of patients following treatment. Given these results, currently SBRT can be considered in patients with CP-A/B and ≤2 tumor nodules measuring ≤10 cm and/or PVT (84); however, it should be used cautiously in the setting of lower platelet counts (OR, 0.90; median, 108 × 10^9^/L vs. 150 × 10^9^/L) or higher dose to 800 cc of liver (OR, 1.11; median, 14.3 Gy vs. 6.0 Gy) as these factors are strongly associated with liver function decline (150). Finally, multiple comparative trials are currently ongoing to define optimal sequencing of SBRT in combination with other treatment modalities (NCT02323360, NCT02182687, NCT02762266, NCT02470533, and RTOG 1112).

### Proton Beam Therapy

Protons, unlike photons, exhibit a sharp dose falloff known as the Bragg peak (151). The lack of exit dose with PBT becomes important in the management of HCC as it allows the sparing of large volumes of normal liver parenchyma and other surrounding OAR, which may potentially decrease the risk of toxicity while permitting possible escalation of radiation doses.

Numerous single-institutional series have evaluated the efficacy and toxicity of PBT for HCC (Table 6) (104–110). Nakayama et al. reported outcomes in a prospective study of 318 patients with HCC (74% CP-A, 24% CP-B, ≤3 tumor nodules, and 14% PVT) treated with 55–79.2 Cobalt Gy Equivalent (CGE) in 10–35 fractions from 2001 to 2007 (111). The 1-year and 5-year OS rates were 90% and 45%, respectively, and only 1.6% of patients experienced grade ≥3 toxicity. Fukuda et al. recently published 5-year clinical outcomes in a prospective study of 129 patients with HCC (78% CP-A, 22% CP-B, 92% ≤2 tumor nodules, and 61% >3 cm tumors) treated with 66–70 CGE in 10–35 fractions from 2002 to 2009 (152). The 5-year local tumor control and OS rates were 94% and 69% for patients with BCLC 0/A stage, 87% and 66% for patients with BCLC B stage, and 75% and 25% for patients with BCLC C stage disease. No grade ≥3 toxicity was observed. Based on these data, PBT is considered in CP-A/B patients with ≤3 tumor nodules measuring >3 cm and/or PVT.

**Table 6:** Studies of proton beam radiation therapy for hepatocellular carcinoma.

References	Study design	Modality	*N*	Tumor size	CPS A/B/C (%)	Radiation therapy Dose	Follow up	Local control	Overall survival
Bush et al. (104)	Prospective	Proton	76	5.5 cm	29/47/24	63 GyE in 15 fx	90 mo	5 yr 80%	3 yr 70%
Hong et al. (105)	Prospective	Proton	44	5 cm (1.9–12)	73/27/0	58 GyE in 15 fx	81 mo	2 yr 94.8%	2 yr 63%
Fukumitsu et al. (106)	Prospective	Proton	51	2.8 cm	81/19/0	66 GyE in 10 fx	110 mo	3 yr 95%	3 yr 42%
				(0.8–9.3)				5 yr 89%	5 yr 39%
Mizumoto et al. (107)	Prospective	Proton	53	4.3 cm	87/11/2	72.6 GyE in 22 fx	NR	2 yr 94%	2 yr 57%
				(1–13)				3 yr 86%	3 yr 45%
Kim et al. (108)	Prospective	Proton	27	All ≤ 7 cm	89/11/0	60–72 GyE in 20-24 fx	31 mo	3 yr 80%	3 yr 56%
								5 yr 64%	5 yr 42%
Nakayama et al. (109)	Prospective	Proton	47	NR	74/19/7	72.6–77 GyE in 22–35 fx	23 mo	1 yr 92%	1 yr 70%
								3 yr 88%	3 yr 50%
								4 yr 88%	4 yr 34%
Sugahara et al. (110)	Prospective	Proton	22	11 cm	50/50/0	47.3–89 GyE in 10–35 fx	13 mo	2 yr 87%	1 yr 64%
				(10–14)					2 yr 36%
Nakayama et al. (111)	Retrospective	Proton	318	NR	74/24/2	55–77 GyE in 10–35 fx	19 mo	NR	1 yr 90%
									3 yr 65%
									5 yr 45%

*CPS, Child-Pugh score; NR, not reported; C/P res, complete/partial response*.

For relatively large tumors located near OAR, delivering tumoricidal doses of RT while sparing normal tissue becomes more challenging. “Dose painting,” or simultaneous integrated boost (SIB), typically used in the setting of IMRT or PBT, allows for the delivery of different RT doses to different regions at the same time. The gross tumor volume can receive higher doses, while areas of subclinical disease abutting OAR (e.g., GI tract) can receive lower doses. Kim et al. retrospectively reported favorable outcomes among patients with inoperable HCC who underwent SIB-PBT. Forty-one patients with tumor vascular thrombosis (93% CP-A, 61% >5 cm tumor, range 2–16 cm) received 50 CGE, 60 CGE or 66 CGE in 10 fractions to planning target volume 1 (and 30 CGE to planning target volume 2) based on the distance between the gross tumor volume and GI tract (<1 cm [*n* = 27], 1–1.9 cm [*n* = 7], or ≥2 cm [*n* = 7]) (153). Two-year OS and local PFS rates were 51.1% and 88.1%, respectively. There were no grade ≥3 toxicities. This retrospective study provides promising results with a novel treatment delivery technique in patients with relatively large tumors. While dosimetric studies demonstrated that PBT can better spare the liver compared to photon therapy, it remains unclear if this translates to a clinically relevant decrease in hepatotoxicity.

### TACE Plus RT

Historically, the use of RT for HCC was limited by the risk of RILD; however, as described above, with advances in EBRT delivery techniques, utilization of RT has increased and can be used in conjunction with other therapies including TACE. Thirty prospective trials, primarily from China, demonstrated that TACE plus RT significantly improves OS compared to TACE alone (Table 7) (112–141). A recent meta-analysis evaluated 25 trials involving 2,577 patients treated with TACE plus RT or TACE alone (154). This meta-analysis revealed TACE plus RT significantly improved 1-, 2-, 3-, 4-, and 5-year OS rates as well as complete and partial tumor response in patients with unresectable HCC (respectively: OR, 1.36 [95% CI, 1.19–1.54]; OR, 1.55 [95% CI, 1.31–1.85]; OR, 1.91 [95% CI, 1.55–2.35]; OR, 3.01 [95% CI, 1.38–6.55]; OR, 3.98 [95% CI, 1.86–8.51]). While TACE plus RT improved OS, it also significantly increased the risk of gastroduodenal ulcers (OR, 12.80 [95% CI, 1.57–104.33]), and caused elevations in ALT (OR, 2.46 [95%CI, 1.30–4.65]) as well as total bilirubin levels (OR, 2.16 [95% CI, 1.05–4.45]) when compared to TACE alone. Therefore, appropriate patient selection is important to avoid the possibility of added toxicity.

**Table 7:** Prospective studies comparing transcatheter arterial chemoembolization plus radiation therapy with transcatheter arterial chemoembolization alone for hepatocellular carcinoma.

**References**	***N***	**CPS A/B/C (%)**	**Tumor size**	**Chemotherapy**	**RT dose**	**Modality**	**Follow up**	**TACE & RT interval**	**Overall survival**	***p***
									**TACE + RT vs. TACE alone**	
Liao et al. (112)	48	71/29/0	3.5–11 cm	5-Fu (1.0–1.25 g) + DDP (70–90 mg) + ADM (50–60 mg)	40–66 Gy in 20–33 fx	3D-CRT	3 yrs	1–2 wks	1 yr 74% vs.50% 3 yr 30% vs.14%	0.036
Zhao et al. (113)	96	100/0/0	All <6cm	5-Fu (7.5 g) + HCPT (15 mg) + DDP (40 mg)	45–55 Gy @ 4–5 Gy/fx	3D-CRT	3 yrs	3 wks	1 yr 82% vs.52% 2 yr 63% vs.28% 3 yr 43% vs.15%	<0.05
Li et al. (114)	82	61/39/0	3.2–11.5 cm	5-Fu (1.0–1.25 g) + HCPT (20–30 mg) + DDP (60–80 mg) + MMC (14–20 mg) + EPI (50–60 mg)	36–56 Gy @ 4–8 Gy/fx	3D-CRT	3 yrs	4–6 wks	1 yr 73% vs.55% 2 yr 59% vs.27% 3 yr 42% vs.13%	<0.05
Peng et al. (115)	91	NA	43% of the patients with >10 cm	DDP (20–40 mg) + ADM (40–80 mg) + MMC (10–20 mg) + 5-Fu (1.0–1.25 g)	40–50 Gy in 34–42 fx	3D-CRT	5 yrs	4–6 wks	1 yr 73% vs.52% 2 yr 36% vs.12% 3 yr 29% vs.5%	<0.05
Leng et al. (116)	75	100/0/0	10 cm (median)	5-Fu (1.0–2.0 g) +DDP (60–120 mg) + ADM (50–100 mg)	45–60 Gy @ 4.8–7 Gy/fx	NR	3 yrs	4–8 wks	1 yr 75% vs.61% 2 yr 57% vs.34% 3 yr 40% vs.20%	<0.05
Liu et al. (117)	114	73//27/0	27% of the patients with ≥10 cm	EPI (50–60 mg) + MMC (14–20 mg) + CBP (300 mg)	36–60 Gy @ 1.8–2 Gy/fx	3D-CRT	3 yrs	4–8 wks	1 yr 67% vs.54% 2 yr 48% vs.37% 3 yr 37% vs.19%	<0.05
Shang et al. (118)	76	100/0/0	All <6 cm	5-Fu (1.0g) + DDP (40–60 mg) + EPI-ADM (60 mg) + MMC (10–20 mg)	≤30 Gy @ 2 Gy/fx	3D-CRT	3 yrs	3 wks	1 yr 78% vs.50% 2 yr 60% vs.32% 3 yr 34% vs.18% 2 yr 54% vs.33%	<0.05
Wang et al. (119)	40	85/15/0	NR	DDP (60 mg) + ADM (40 mg) + MMC (10 mg) or FUDR (1.0 g)	50 Gy @ 1.5–1.8 Gy/fx	Moving strip	5 yrs	2 wks	6 mo 70% vs.60% 1 yr 40% vs.25%	<0.05
Zhang et al. (120)	259	100/0/0	2.2–16.4 cm	5-Fu (0.5–1.0g) + EPI (30–50 mg) + CBP (200–300 mg) + ADM (10–20 mg)	36–50 Gy @ 3–5 Gy/fx	3D-CRT	2 yrs	5–30 days	1 yr 86% vs.65% 2 yr 25% vs.15% 3 yr 15% vs.5% 5 yr 10% vs.0%	<0.05
Xiao et al. (121)	60	65/35/0	2.5–16.0 cm	5-Fu (0.75 g) + HCPT (15 mg) + DDP (40 mg)	55 Gy @ 5 Gy/fx	3D-CRT	3 yrs	1–3 wks	1 yr 87% vs.53% 2 yr 53% vs.34% 3 yr 33% vs.17%	<0.01
Kang et al. (122)	120	87/13/0	All ≤ 10 cm	5-Fu (1.25 g) + HCTP (20 mg) + DDP (60 mg) + MMC (14 mg)	50–60 Gy @ 2 Gy/fx	3D-CRT	3 yrs	4 wks	1 yr 75% vs.56% 2 yr 62% vs.31% 3 yr 44% vs.15%	<0.05
Yang et al. (123)	61	NR	All ≤ 10 cm	5-Fu (1.25 g) + HCPT (20 mg) + DDP (60 mg) + MMC (14 mg)	40–50 Gy @ 5 Gy/fx	3D-CRT	3 yrs	4 wks	1 yr 68% vs.53% 2 yr 55% vs.30% 3 yr 39% vs.18%	<0.05
Wu et al. (124)	207	NR	82% of the patients with ≥5 cm	5-Fu (1.0 g) + HCPT (15 mg) + EPI (50 mg) + DDP (60 mg)	38–63 Gy in 7–15 fx	3D-CRT	3 yrs	3–4 wks	1 yr 79% vs.55% 2 yr 48% vs.24% 3 yr 31% vs.6%	<0.05
Yubing et al. (125)	54	NR	44% of the patients with >5 cm	DDP (60 mg) + Tegafur (1.0 g) + EPI (40 mg) + MMC (10 mg)	54–60 Gy @ 2 Gy/fx	3D-CRT	3 yrs	2 wks	1 yr 74% vs.59% 2 yr 44% vs.33% 3 yr 30% vs.19%	<0.05
Guo et al. (126)	114	71/29/0	66% of the patients with ≥10 cm	5-Fu (1.0 g) + DDP (40–60 mg) + MMC (10–20 mg) + ADM (40 mg)	30–50 Gy @ 1.8–2 Gy/fx	NR	3 yrs	NR	1 yr 72% vs.52% 2 yr 49% vs.17% 3 yr 45% vs.7%	<0.05
Cai et al. (127)	94	NR	All <6 cm	5-Fu (1.0 mg) + DDP (40 mg) or HCPT (15 mg) + EPI (60–80 mg)	45–55 Gy **@** 4–5 Gy/fx	3D-CRT	3 yrs	3 wks	1 yr 83% vs.52% 2 yr 62% vs.25% 3 yr 43% vs.15%	<0.05
Tan et al. (128)	87	73/27/0	61% of the patients with >5 cm	DDP (40–60 mg) + ADM (30 mg) + MMC (6–10 mg) + 5-Fu (1.0 mg)	48–60 Gy @ 2.6–3.2 Gy/fx	3D-CRT	3 yrs	4–6 wks	1 yr 73% vs.59% 2 yr 53% vs.31% 3 yr 36% vs.14%	<0.05
Xie et al. (129)	122	NR	All <6 cm	5-Fu (0.75 g) +HCPT (15 mg) + DDP (40 mg)	45–55 Gy @ 4–5 Gy/fx	3D-CRT	3 yrs	3 wks	1 yr 85% vs.59% 2 yr 65% vs.30% 3 yr 39% vs.18%	<0.05
Wang et al. (130)	108	100/0/0	38% of the patients with ≥5 cm	5-Fu (1.0 g) + ADM (50 mg) + DDP (60 mg)	45–60 Gy @ 5.8–7 Gy/fx	NR	3 yrs	3–6 wks	1 yr 77% vs.53% 2 yr 57% vs.32% 3 yr 42% vs.19%	<0.05
Zhan et al. (131)	44	75/25/0	NR	DDP (60 mg) + ADM (40–60 mg) + GEMn (1.2 g) + 5-Fu (1.0 g)	50–60 Gy @ 2–3 Gy/fx	3D-CRT	2 yrs	3 wks	1 yr 73% vs.46% 2 yr 50% vs.18%	<0.05
Liu et al. (132)	50	66/44/0	6.6 cm (4–12)	DDP (40–60 mg) + EPI (60–80 mg) + 5-Fu (1.25 g) + MMC (10–20 mg)	45–54 Gy @ 3 Gy/fx	3D-CRT	2 yrs	3–5 wks	1 yr 76% vs.48% 2 yr 50% vs.24%	<0.05
Wang et al. (133)	60	80/20/0	5.8 cm (4.5–11)	DDP (60–80 mg) + EPI (60–80 mg) + 5-Fu (1.0 g)	42–53 Gy @ 4–6 Gy/fx	3D-CRT	2 yrs	4–6 wks	1 yr 80% vs.53% 2 yr 47% vs.27%	<0.05
Rui-wen et al. (134)	45	NR	All <10 cm	DDP (60 mg) + MMC (14 mg) + 5-Fu (1.25 g) + HCPT (20 mg)	40–45 Gy in 5 fx	3D-CRT	3 yrs	4 wks	1 yr 78% vs.51% 2 yr 60% vs.24% 3 yr 41% vs.15%	<0.05
Lan et al. (135)	102	NR	All >3 cm	5-Fu (0.5–1.0 g) + DDP (40–80 mg) + HCPT (20–40 mg)	51.3–69 Gy @ 1.15–1.4 Gy/fx	3D-CRT	3 yrs	4–6 wks	1 yr 57% vs.26% 3 yr 62% vs.17%	<0.05
Guo et al. (136)	76	83/17/0	All >5 cm	5-Fu (1.0 g) + DDP (40–60 mg) or ADM (30–50 mg)	30–50 Gy @ 1.8–2 Gy/fx	3D-CRT	26 mo	4–8 wks	1 yr 64% vs.40% 3 yr 29% vs.10% 5 yr 19% vs.7%	0.0001
Song et al. (137)	56	39/61/0	9.2 cm	5-Fu (1.0 g) + DDP (40–60 mg) + ADM (40–50 mg) + MMC (12–16 mg)	39–58.5 Gy @ 3 Gy/fx	Moving strip	3 yrs	2–3 wks	1 yr 72% vs.52% 2 yr 58% vs.39% 3 yr 40% vs.21%	<0.05
Zhang et al. (138)	96	95/5/0	NR	EPI (30–50 mg) + 5-Fu (1.0–1.5 g) +/or DDP (40–60 mg)	30–50 Gy @ 5–12 Gy/fx	SBRT	3 yrs	4 wks	2 yr 41% vs.29% 3 yr 32% vs.23%	<0.05
Chen et al. (139)	158	NR	27.5 cm	5-Fu (0.75–1.0 g) + DDP (40–60 mg) + farmorubicin (40–80 mg) + MMC (6–10 mg)	50–62 Gy @ 2–2.5 Gy/fx	3D-CRT	3 yrs	2 wks	1 yr 79% vs.59% 2 yr 55% vs.36% 3 yr 26% vs.16%	<0.05
Shim et al. (140)	73	90/10/0	10 cm	ADM (20–50 mg)	30.6–59.4 Gy @ 1.8 Gy/fx	3D-CRT	2 yrs	7–10 days	1 yr 70% vs.33% 2 yr 37% vs.14%	0.001
Zhang et al. (141)	52	NR	NR	Oxaliplatin (100 mg) + EPI (30 mg)	40–50 Gy @ 4–5 Gy/fx	3D-CRT	1 yr	4–5 wks	1 yr 72% vs.44%	<0.05

*CPS, Child-Pugh score; 3D-CRT, 3D-conformal radiotherapy; TACE, transarterial chemoembolization; NR, not reported; DDP, Cisplatin; 5-Fu, 5-fluorouracil; ADM, adriamycin; MMC, mitomycin C; HCPT, 10-Hydroxycamptothecin; EPI, epirubicin; FUDR, floxuridine; CBP, carboplatin; GEM, gemcitabine; SBRT, stereotactic body radiotherapy*.

TACE plus RT significantly improves survival compared to TACE alone or RT alone based on randomized studies detailed in Table 7. Leng et al. prospectively randomized 107 unresectable HCC patients with CP-A (median tumor size 10 cm) to RT alone, TACE alone, or TACE plus RT (116). The 1-, 2-, and 3- year OS rates were significantly higher with TACE plus RT (75%, 57%, and 40%) compared to TACE alone (61%, 34%, 20%) and RT alone (53%, 31%, and 19%) (*p* < 0.05). Liu et al. randomized 50 HCC patients with ≤2 tumor nodules (66% CP-A, 44% CP-B, and median tumor size 6.6 cm, range 4–12 cm) to TACE plus RT or TACE alone (132). The 1- and 2-year OS rates were significantly higher with TACE plus RT (76% and 56% vs. 48% and 24%, *p* < 0.05). Wang et al. randomized 60 HCC patients (80% CP-A, 20% CP-B, 92% ≤3 tumor nodules, and median tumor size 5.8 cm, range 4.5–11 cm) to TACE plus RT or TACE alone (133). The 1- and 2-year OS rates were significantly higher with TACE plus RT (80% and 47%) compared to TACE alone (53% and 27%) (*p* < 0.05). Finally, in the setting of large volume disease, a single nodule >15 cm or nodules totaling a maximum sum of <20 cm, TACE plus RT is a treatment option given the significant survival benefit compared to TACE alone (1-, 2-, 3- year OS 79% vs. 59%, 55% vs. 36%, and 26% vs. 16%, *p* = <0.05) (139). In light of this data, TACE plus RT must be considered in CP-A and B disease patients with ≤3 tumor nodules measuring ≥3 cm.

Non-randomized data suggests TACE plus RT improves outcomes for HCC patients with partial PVT (155, 156). More recently, Yoon et al. randomized 90 HCC patients with macroscopic vascular invasion to TACE plus RT (87% multinodular disease, measuring 9.8 cm, range 8–13 cm) or sorafenib (78.9% multiple lesions, median maximal tumor diameter 9.7 cm, range 7–12 cm) between 2013 and 2016 (157). All patients had tumor portal vein invasion and CP-A liver function. The TACE plus RT group experienced a significantly longer median TTP (31 weeks vs. 12 weeks, *p* < 0.01) and median OS (55 weeks vs. 43 weeks, *p* = 0.04) than the sorafenib group. Furthermore, 11% of patients treated with TACE plus RT had curative surgery due to downstaging. Therefore, the combination of TACE and RT can be considered a treatment option in patients with partial PVT.

In the setting (4–6 nodules) of multinodular HCC, TACE plus RT also improves survival in CP-A and B disease. Peng et al. randomized 91 patients to TACE plus RT (23% multinodular disease) or TACE alone (17% multinodular disease) (115). The 1- and 3-year OS rates were significantly higher with TACE plus RT (73% and 36%) compared to TACE alone (52% and 12%) (*p* < 0.05). Yubing et al. randomized 54 patients to TACE plus RT (63% multinodular disease) or TACE alone (56% multinodular disease) (125). The 1- and 3-year OS rates were significantly higher with TACE plus RT (74% and 30%) compared to TACE alone (60% and 19%) (*p* < 0.05). Guo et al. randomized 114 patients (71% CP-A, 29% CP-B, and 37% multinodular disease) to TACE plus RT or TACE alone (126). The 1- and 3-year OS rates were significantly higher with TACE plus RT (72% and 45%) compared to TACE alone (52% and 17%) (*p* < 0.05). There were no differences in toxicities between treatment groups, other than a more frequent elevation in alanine aminotransferase levels with TACE plus RT (Grade ≤ 2, 25% with combination vs. 7% with TACE alone). In light of this prospective data, TACE plus RT should be considered in patients with multinodular HCC.

## Conclusions

While the BCLC classification provides a framework for treatment selection of patients with HCC, it may simplify the decision-making process and may not uniformly take into consideration recent studies, and detailed tumor volumes, which may better guide decisions about local therapies, including radiation therapy and combination treatments. We critically reviewed the literature and devised a data-driven treatment algorithm for optimizing outcomes for patients with unresectable BCLC Stage B or C HCC (Figure 1). This treatment algorithm captures modern data to guide treatment options for those with CP-A or B and ≤3 nodules measuring >3 cm, multinodular disease, or PVT, incorporating tumor volume considerations.

For unresectable, localized HCC patients with either partial PVT, ≤3 nodules >3 cm or multinodular disease, prospective, randomized data suggest that TACE plus RT provides improved survival outcomes and response rates compared to TACE alone; however, toxicities appear more frequent with combination therapy (154). Therefore, appropriate patient selection is important to minimize toxicity. The treatment algorithm used at our institution is shown in Figure 1. For patients with PVT (either partial or complete), RT alone (delivered via IMRT, SBRT or PBT) appears to be a viable option for those unfit to undergo TACE plus RT. One-year survival rates of 44–69% and 30–51% have been observed for those with partial and complete PVT, respectively. For patients without PVT who have ≤3 nodules measuring >3 cm, TACE plus RT results in 1-year survival rates of 75–85%. Lastly, for patients without PVT who have multinodular disease, TACE alone or TARE with ^90^Y is a reasonable option for those unfit for TACE plus RT, particularly those presenting with ≥7 nodules. One-year survival rates of 46–70% have been observed after either modality.

We acknowledge that the best treatment approach is determined through a multidisciplinary management approach in experienced cancer centers with a dedicated HCC program. This offers robust access to all the modalities discussed plus systemic therapy. We hope that this data driven treatment algorithm will aid clinicians in managing localized HCC.

## Author Contributions

All authors listed have made a substantial, direct and intellectual contribution to the work, and approved it for publication.

### Conflict of Interest Statement

The authors declare that the research was conducted in the absence of any commercial or financial relationships that could be construed as a potential conflict of interest.
